# The relationship between islet β-cell function and metabolomics in overweight patients with Type 2 diabetes

**DOI:** 10.1042/BSR20221430

**Published:** 2023-02-02

**Authors:** You Lv, Yuanyuan Zheng, Xue Zhao, Zhuo Li, Guixia Wang

**Affiliations:** 1 Department of Endocrinology and Metabolism, The First Hospital of Jilin University, Changchun, Jilin, China; 2 Department of Geriatric Cardiovascular, The Second Affiliated Hospital of Xi’an Jiaotong University (Xibei Hospital), Xi’an, Shanxi, China

**Keywords:** β cell function, insulin resistance, metabolomics, overweight, Type 2 diabetes

## Abstract

A cross-sectional study was performed using metabolomics in overweight patients with Type 2 diabetes (T2D) at different stages of the disease. We aimed to identify potential metabolites for assessing islet β-cell function in order to investigate the correlation between islet β-cell dysfunction and metabolite changes in overweight patients with T2D. We selected 60 overweight adults (24 ≤ body mass index [BMI] < 28 kg/m^2^) with T2D who had been admitted to our hospital. The participants were equally divided into three groups according to disease duration: H1 (duration ≤ 5 years), H2 (5 years < duration ≤ 10 years), and H3 (duration > 10 years). Questionnaires, physical examinations, laboratory tests, and imaging studies were administered to all participants. The modified homeostasis model of assessment (HOMA) index was calculated using fasting C-peptide levels, and metabolite assays were performed using mass spectrometry. The results showed that HOMA-β and visceral fat area (VFA) were negatively correlated with diabetes duration. The VFA was positively correlated with arginine, cysteine, methionine, proline, and succinyl/methylmalonylcarnitine levels. The HOMA-β was negatively correlated with the serine and tetradecanoyldiacylcarnitine levels, and positively correlated with the aspartic acid, cysteine, homocysteine, piperamide, proline, and valine levels. The HOMA-IR was negatively correlated with hydroxypalmitoylcarnitine levels and positively correlated with the myristoylcarnitine levels. Thus, at different stages of T2D progression in overweight patients, serine, aspartic acid, cysteine, homocysteine, piperamide, proline, valine, and tetradecanoyldiacylcarnitine may be associated with HOMA-β and represent potential novel biomarkers for evaluating islet β-cell function.

## Introduction

Due to economic development, lifestyle changes, and the rapidly aging population, China shows the highest incidence of diabetes mellitus (DM) in the world. According to the guideline for the prevention and treatment of Type 2 DM in China (2020 edition), the prevalence of DM rose from 0.67% in 1980 to 11.2% in 2017 [1]. In 2018, the estimated overall prevalence of diabetes in China was 12.4% and that of prediabetes was 38.1% [2]. DM is a major public health problem in China. The number of overweight and obese Chinese people has increased rapidly in recent years; similarly, the prevalence of DM among overweight and obese people has increased markedly. In 2013, the prevalence of DM was 7.8% in individuals with BMI < 25 kg/m^2^, 15.4% in those with 25 kg/m^2^ ≤ BMI < 30 kg/m^2^, and 21.2% in those with BMI ≥ 30 kg/m^2^ [1,3]. Obesity plays an important role in the development of insulin resistance and DM [4–6], and is associated with microvascular complications in patients with Type 2 diabetes (T2D) [7]. The mechanisms underlying the relationship between obesity and DM are not yet fully understood [8].

Metabolomics focuses on testing and quantitatively analyzing small-molecule metabolites. In recent years, metabolomics has become increasingly popular as a clinical tool for assessing disease risk. Many studies have investigated the changes in metabolite biomarkers in patients with DM and obesity, substantially contributing to the understanding of the pathophysiological processes of metabolic diseases [9].

In 1984, Nicholson et al. used nuclear magnetic resonance technology to compare the urine and blood samples from control and diabetic groups and found significant differences between the metabolites in these samples [10]. Since then, an increasing number of studies have identified several metabolic regulatory pathways in DM. Techniques based on gas chromatography-mass spectrometry (GC-MS) and liquid chromatography-MS (LC-MS) have been used to investigate the profiles of various metabolites in healthy individuals with T2D. The metabolism of amino acids, fatty acids, glycerophospholipids, and sphingomyelin (SM) in patients with diabetes or with impaired fasting glycemia (IFG) are abnormal. Metabolic markers, such as alanine, proline, and glutamine, have been found in these patients [11]. Suhre et al. identified metabolites that were used to detect diabetes-related complications under subclinical conditions [12]. Newgard et al. found that there were differences in the plasma concentrations of branched-chain amino acids (BCAAs: leucine, isoleucine, and valine), methionine, glutamate/glutamine (Glx), aromatic amino acids (phenylalanine and tyrosine), and C3 and C5 acylcarnitines between obese subjects and lean subjects [13]. Metabolomics is a potential noninvasive tool for the early diagnosis of T2D, and can provide new insights into the pathophysiological mechanisms underlying this condition [14].

The pathophysiological characteristics of T2D include insulin resistance and islet β-cell dysfunction. Islet β-cell function gradually deteriorates as the disease progresses. However, the current methods used to evaluate β-cell function are not accurate in clinical practice. Many metabolomics studies have found that amino acids, fatty acids, and other small molecular metabolites are involved in the occurrence and development of insulin resistance and islet β-cell dysfunction. Thus, metabolomics is becoming a novel research approach to evaluate islet β-cell function.

In this cross-sectional study, we investigated the relationship between islet β-cell function and metabolite changes in overweight patients with T2D at different disease stages. We also explored potential metabolite markers for evaluating islet β-cell function in clinical practice.

## Materials and methods

### Patients

Based on the inclusion criteria listed below, 60 overweight patients with T2D were included in the present study at the First Hospital of Jilin University in Changchun, China. The 60 patients were divided into three groups, according to the duration of diabetes: H1 group, disease duration ≤ 5 years; H2 group, 5 years < disease duration ≤ 10 years; H3 group, disease duration > 10 years. All participants provided signed written consent, and the study protocol was approved by the Ethical Committee of The First Hospital of Jilin University.

Inclusion criteria:

1. T2D according to the 1999 WHO diagnostic criteria for diabetes
2. 18–75 years old
3. Overweight, 24 ≤ BMI < 28 kg/m^2^ (expert consensus and standard on weight management for overweight or obese people in China)

Exclusion criteria:

1. Type 1 diabetes, gestational diabetes and diabetes with pregnancy, patients with diabetes after pancreatitis and pancreatectomy, and special types of diabetes.
2. Acute diabetic complications.
3. Acute cardiovascular or neurological diseases.
4. Liver and kidney failure and malignant tumors.
5. Other endocrine diseases which can affect islet β-cell function and insulin sensitivity, such as pituitary tumors, Graves’ disease, etc.

### Anthropometry material

Personal information, medical history, physical examination, and laboratory results were collected and an HOMA index was calculated. The information is presented in Table 1.

**Table 1:** Clinical characteristics of participants

Characteristic	H1 (*n*=20)	H2 (*n*=20)	H3 (*n*=20)	*F*/*X*^2^	*P* value
Gender (men/women)	(18/2)	(13/7)	(14/6)	3.733	0.155
Family history of diabetes (yes/no)	(5/15)	(10/10)	(10/10)	3.429	0.180
Smoking (yes/no)	(12/8)	(7/13)	(9/11)	2.545	0.280
Drinking (yes/no)	(8/12)	(7/13)	(10/10)	0.960	0.619
Hypertension (yes/no)	(5/15)	(6/14)	(13/7)*	7.917	0.019
Coronary heart disease (yes/no)	(2/18)	(0/20)	(3/17)	3.055	0.217
Cerebral infarction (yes/no)	(3/17)	(1/19)	(4/16)	2.019	0.364
Hyperlipemia (yes/no)	(11/9)	(6/14)	(8/12)	2.606	0.272
Fatty liver (yes/no)	(7/13)	(6/14)	(13/7)	5. 837	0.054
Hyperuricemia (yes/no)	(2/18)	(4/16)	(2/18)	1.154	0.562
Diabetic peripheral neuropathy (yes/no)	(9/11)	(11/9)	(12/8)	0.938	0.626
Diabetic retinopathy (yes/no)	(3/17)	(4/16)	(8/12)	3.733	0.155
Age	48.85 ± 12.94	56.25 ± 12.15	60.35 ± 10.39*	4.82	0.012
Disease duration (year)	1.82 ± 1.87	7.65 ± 1.46*	15.75 ± 3.02*	52.451	0.000
BMI (kg/m^2^)	25.78 ± 1.22	26.33 ± 1.03	25.85 ± 1.1	1.46	0.242
WHR	0.94 ± 0.04	0.93 ± 0.06	0.93 ± 0.04	0.22	0.806
Systolic pressure (mmHg)	121.5 (115, 131.75)	127.5 (113.25, 146.75)	128 (115, 135.5)	0.126	0.939
Diastolic pressure (mmHg)	84.7 ± 10.7	81.75 ± 12.57	82.5 ± 12.43	0.33	0.720
VFA (cm^2^)	127.4 ± 20.37	112.1 ± 19.04*	107.1 ± 30.68*	3.91	0.026
SFA (cm^2^)	169.42 ± 42.71	174.1 ± 34.1	181.7 ± 37.68	0.52	0.596
HOMA-IR	3.88 (3.21, 5.47)	3.24 (2.86, 4.91)	3.39 (2.66, 3.64)	3.265	0.195
HOMA-β	79.57 (36.31, 126.34)	52.22 (35.87, 73.29)	45.08 (27.17, 68.54)*	6.975	0.031
FBG (mmol/L)	7.51 ± 2.24	8.05 ± 2.5	8.11 ± 2.27	0.40	0.675
2hPG (mmol/L)	12.28 ± 3.61	11.57 ± 2.9	11.67 ± 2.74	0.31	0.736
FCP (nmol/L)	1.04 ± 0.44	0.82 ± 0.41	0.74 ± 0.4	2.74	0.073
HbA1c (%)	8.26 ± 1.99	8.17 ± 1.81	8.44 ± 1.57	0.12	0.886
Proteinuria (g/24 h)	0.24 (0.1, 0.3)	0.17 (0.13, 0.24)	0.24 (0.18, 0.5)	5.342	0.069
Urine microalbumin (mg/24 h)	10.8 (10.8, 49.2)	10.8 (10.8, 19.52)	10.8 (10.8, 183.55)	3.021	0.221
Urinary protein/creatinine ratio (mg/mmol)	0.02 (0.02, 0.08)	0.03 (0.02, 0.05)	0.03 (0.02, 0.14)	2.592	0.274
TG (mmol/L)	3.28 (1.74, 7.96)	2.18 (1.32, 2.9)	2.13 (1.52, 2.64)	5.763	0.056
TC (mmol/L)	5.31 ± 2	4.54 ± 0.84	4.23 ± 0.85	5.025	0.081
LDL (mmol/L)	2.62 (2, 3.71)	2.89 (2.53, 3.54)	2.47 (2.08, 2.79)	4.567	0.102
HDL (mmol/L)	1.02 ± 0.24	1.08 ± 0.23	1.08 ± 0.29	0.34	0.711
γ-GT (U/L)	40.85 (29.9, 68.73)	36.6 (29.13, 52.25)	34.25 (27.25, 39.98)	1.423	0.491
AKP (U/L)	76.12 ± 26.31	77.13 ± 19.1	73 ± 25.06	0.17	0.848
ALT (U/L)	22.75 (13.38, 35.25)	25.3 (19.2, 48.35)	18.15 (15.23, 25.1)	3.011	0.222
AST (U/L)	23.55 (18.45, 31.98)	24.75 (21, 33.98)	19.4 (17.53, 26.2)	3.981	0.137
BUN (mmol/L)	6.67 ± 2.57	6.75 ± 1.73	6.21 ± 2.02	0.36	0.698
Scr (umol/L)	67.4 ± 17.94	70.21 ± 12.5	75.17 ± 19.13	1.10	0.341
UA (umol/L)	394.02 ± 136.79	353.6 ± 91.62	354.2 ± 86.12	0.93	0.399

*It compared with H1 group, *P*<0.05; It compared with H2 group, *P*<0.05.

**Abbreviations**: γ-GT, γ-glutamyl transpeptidase; 2hPG, 2-h postprandial blood glucose; ALP, alkaline phosphatase; ALT, alanine aminotransferase; AST, aspartate aminotransferase; BUN, blood urea nitrogen; FBG, fasting blood glucose; FCP, fasting C-peptide; HbA1c, glycosylated hemoglobin; HDL, high-density lipoprotein cholesterol; LDL, low-density lipoprotein cholesterol; Scr, serum creatinine; TC, total cholesterol; TG, triglyceride; UA, uric acid.

HOMA index: HOMA-IR (CP) = 1.5 + fasting blood glucose × fasting C-peptide/2800; HOMA-β (CP-DM) = 0.27 × fasting C-peptide/(fasting blood glucose-3.5), (C-peptide: pmol/L; blood glucose: mmol/L).

### Metabolomics quantification

#### Chemical reagents

Acetonitrile (HPLC grade, Thermo Fisher), pure water (Thermo Fisher), *n*-butanol, acetyl chloride (Sigma-Aldrich), NSK-A, and NSK-B isotope internal standard (Cambridge). All standard products were mixed and dissolved in 2 ml pure methanol and stored at 4°C. The working liquid was obtained by diluting 100 times to extract metabolites. The amino acids and carnitine quality control (QC) standards were provided by Chromsystems (Grafelfing, Germany).

#### Sample processing

All patients fasted for 8 h before blood sample collection in the early morning. A circle (diameter, 3 mm) was punched from each of the dried blood spot (DBS) paper and placed in 96-well plates (Millipore, Billerica, MA, U.S.A.) for metabolite extraction. Next, 100 μl of the working solution of each sample was added to each well containing a DBS disc. The plate was shaken for 20 min and centrifuged at 1500 ***g*** for 2 min before the filtrate was collected through the lower layer. Four blank holes were randomly selected in each plate, and two low and two high controls were separately placed. The QC and filtrate were blow-dried using pure nitrogen at 50°C. The dried samples were derivatized with a mixture of 60 μl of acetyl chloride and n-butanol (1:9 v/v) at 65°C for 20 min. The derived samples were blow-dried using a previously described method. Each dried sample was redissolved in 100 μl of fresh mobile phase solution for metabolite analysis.

#### Metabolite analysis

Metabolite analysis was performed using an AB Sciex 4000 QTrap system (AB Sciex, Framingham, MA, U.S.A.). The analyst v1.6.0 software (AB Sciex, Framingham, MA, U.S.A.) was used to control the system and collect data, and ChemoView 2.0.2 (AB Sciex, Framingham, MA, U.S.A.) was used for data processing.

### Statistical methods

The SPSS 20.0 software was used for statistical analysis. The Kolmogorov–Smirnov test was used to test whether the data followed a normal distribution. The normal distribution data are presented as the mean ± standard deviation (X ± S), and the non-normal distribution data are represented by M (P25, P75). For the data that followed a normal distribution and had homogeneity of variance, one-way analysis of variance was performed between multiple groups, and the Fisher Least Significant Difference method was used for comparison among groups. The nonparametric rank sum test was used to compare data that did not follow a normal distribution or homogeneity of variance. The count data were expressed as frequencies, and the chi-square test was used to compare the rates. Spearman’s rank correlation was used for correlation analysis. Statistical significance was set at *α* = 0.05, *P*<0.05.

## Result

### Baseline characteristics of patients

Sixty patients were enrolled in this study; their characteristics are presented in Table 1. The prevalence of hypertension was higher in the H3 group than in the other two groups (*P*<0.05, Table 1).

**Figure 1:**
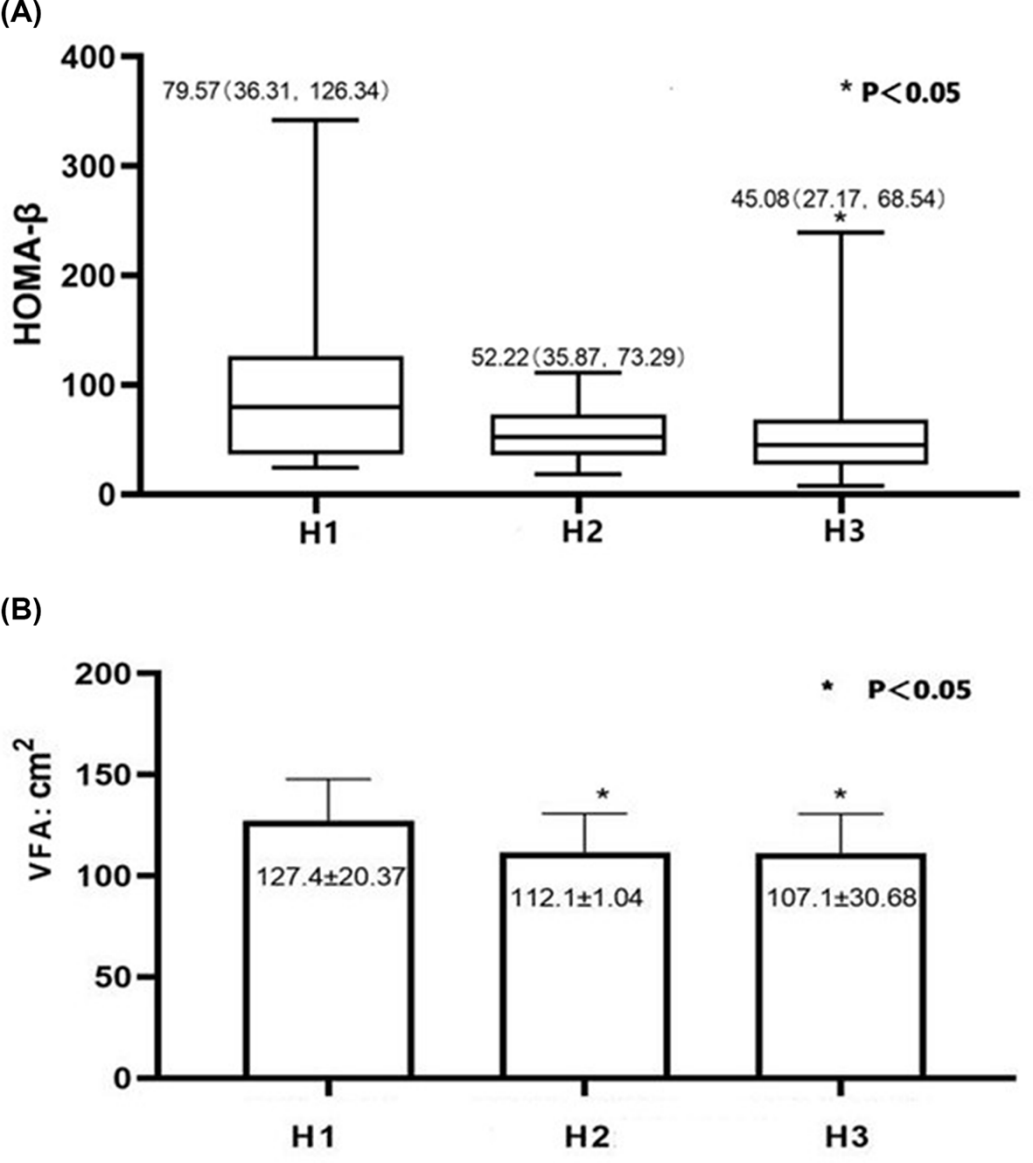
Differences in levels of HOMA-β and VFA in three groups The HOMA-β (**A**) and VFA (**B**) decreased with an increase in disease duration, especially when the disease duration was more than 10 years. * When compared with the H1 group: *P*<0.05.

The HOMA-β was highest in the H1 group, followed by the H2 group, with the H3 group being the lowest. The difference between the H3 and H1 groups was significant (*P*<0.05), but no significant difference was observed between the H1 and H2 groups or the H2 and H3 groups. The VFA was higher in the H1 group than in the other two groups (*P*<0.05, Figure 1). This suggests that insulin sensitivity and VFA decrease with an increase in disease duration.

### Metabolomics analysis

#### Amino acid levels

We tested the levels of 23 amino acids in the present study. Arg, Asp, Gln, His, Pip, and Leu levels in the three groups decreased gradually with diabetes duration (*P*<0.05). Ala levels were lower in the H1 group than in the other two groups (*P*<0.05), and the levels of Cys, Lys, and Pro were higher in the H1 group than in the other two groups (*P*<0.05). Gly, Orn, Val, and Phe levels were the lowest in the H3 group (*P*<0.05). Tyr levels were lower in the H3 group than in the H2 group (*P*<0.05). Ser levels were lower in the H1 group than in the H2 group (*P*<0.05). The levels of the other seven amino acids, Met, Trp, Thr, Asn, Glu, Hcy, and Cit, were not significantly different among the three groups (*P*>0.05, Table 2).

**Table 2:** Amino acid levels

Amino acid	H1 (*n*=20)	H2 (*n*=20)	H3 (*n*=20)	*F*/*X*^2^	*P* value
Ala	149.16 ± 36.35	188.1 ± 44.7*	179.74 ± 42.95*	4.889	0.011
Arg	7.85 ± 3.33	5.61 ± 4.99*	1.69 ± 0.65*	31.778	0.000
Asn	78.06 ± 15.25	86.78 ± 22.17	77.06 ± 14.05	1.859	0.165
Asp	37.42 ± 8.69	25.6 ± 13.32*	17.74 ± 5.63*	25.641	0.000
Cit	19.83 ± 4.9	20.57 ± 5.47	19.22 ± 6.34	0.291	0.749
Cys	2.91 ± 0.9	1.82 ± 1.18*	1.36 ± 0.5*	25.348	0.000
Gln	13.19 ± 4.05	9.49 ± 4.56*	7.15 ± 1.86*	20.617	0.000
Glu	139.8 ± 31.41	123.57 ± 21.94	128.37 ± 32.71	1.643	0.202
Gly	185.59 ± 25.06	180.21 ± 26.21	161.55 ± 25.39*	4.874	0.011
Hcy	8.92 ± 1.01	8.79 ± 0.82	8.7 ± 0.62	0.368	0.694
His	190.5 (167.25,221.5)	45 (42.25,188.25)*	37 (35.5,42.75) *	33.832	0.000
Leu	192.68 ± 34.4	170.19 ± 29.24*	133.36 ± 20.7*	21.816	0.000
Lys	242.85 ± 74.5	182.37 ± 79.71*	144.09 ± 37.39*	11.185	0.000
Met	22.96 ± 4.16	23.65 ± 4.82	20.75 ± 2.83	2.838	0.067
Orn	14.93 ± 2.96	14.53 ± 2.56	11.74 ± 2.54*	8.329	0.001
Phe	39.94 ± 6.98	41.23 ± 6.52	35.05 ± 4.6*	9.911	0.007
Pip	397 (344.75,446)	232.5(173.5,347.5)*	167 (143.25,184.5) *	35.937	0.000
Pro	715.26 ± 207.4	556.06 ± 157.55*	472.33 ± 98.8*	17.606	0.000
Ser	39.69 ± 6.56	45.65 ± 6.53*	41.98 ± 7.43	3.849	0.027
Thr	29.57 ± 6.13	30.3 ± 6.82	26.16 ± 5.94	2.457	0.095
Trp	52.09 ± 13.61	59.5 ± 11.63	52.07 ± 11.25	2.463	0.094
Tyr	53.71 ± 11.5	57.54 ± 10.36	46.35 ± 8.52	6.214	0.004
Val	164.55 ± 21.16	161.15 ± 23.35	129.36 ± 21.18*	15.677	0.000

*It compared with H1 group, *P*<0.05; It compared with H2 group, *P*<0.05.

**Abbreviations**: Ala, alanine; Arg, arginine; Asn, asparagine; Asp, aspartic acid; Cit, citrulline; Cys, cysteine; Gln, glutamine; Glu, glutamic acid; Gly, glycine; Hcy, homocysteine; His, histidine; Leu, leucine; Lys, lysine; Met, methionine; Orn, ornithine; Phe, phenylalaine; Pip, piperamide; Pro, proline; Ser, serine; Thr, threonine; Trp, tryptophan; Tyr, tyrosine; Val, valine.

#### Acylcarnitine levels

We tested the levels of 26 acylcarnitines in the present study. The levels of C4-DC, C4-OH, and C5 in the H1 group were higher than those in the H2 and H3 groups (*P*<0.05). The C4 levels in the H1 group were higher than those in the H3 group (*P*<0.05). The C12 and C14DC levels in the H1 group were lower than those in the H3 group (*P*<0.05). The C14-OH level in the H1 group was higher than that in the H2 group (*P*<0.05). The C5-DC level in the H1 group was lower than that in the other two groups (*P*<0.05). The levels of 18 other acylcarnitines showed no significant differences among the three groups (*P*>0.05, Table 3).

**Table 3:** Acylcarnitine levelsIt should be deleted in all the tables, thanks very much!

Acylcarnitine	H1 (*n*=20)	H2 (*n*=20)	H3 (*n*=20)	*F*/*X*^2^	*P* value
C4	0.21 ± 0.09	0.16 ± 0.05	0.13 ± 0.05*	8.64	0.013
C4DC	0.63 ± 0.25	0.38 ± 0.18*	0.27 ± 0.12*	23.699	0
C4-OH	0.11 (0.09,0.15)	0.06 (0.04,0.07)*	0.05 (0.03,0.06)*	23.928	0
C5	0.16 ± 0.06	0.1 ± 0.04*	0.11 ± 0.04*	11.955	0.003
C5DC	0.05 ± 0.02	0.08 ± 0.04*	0.09 ± 0.04*	10.551	0.005
C12	0.04 (0.03,0.04)	0.04 (0.03,0.06)	0.06 (0.03,0.09) *	9.323	0.009
C14DC	0.02 (0,0.02)	0.02 (0,0.02)	0.02 (0.02,0.02) *	15.131	0.001
C14-OH	0.04 ± 0.02	0.03 ± 0.01*	0.04 ± 0.02	8.691	0.013
C22	0.04 (0.03,0.06)	0.04 (0.04,0.07)	0.05 (0.04,0.09)	6.021	0.05
C0	35.2 ± 8.73	32.11 ± 8.3	28.88 ± 8.51	2.764	0.071
C2	9.75 ± 2.09	10.07 ± 2.79	8.69 ± 2.94	1.496	0.233
C3	1.72 ± 0.65	1.5 ± 0.61	1.28 ± 0.48	2.94	0.061
C5:1	0.06 (0.04,0.08)	0.04 (0.03,0.07)	0.04 (0.03,0.08)	1.106	0.575
C5-OH	0.24 ± 0.09	0.23 ± 0.07	0.2 ± 0.09	1.871	0.163
C6	0.08 ± 0.04	0.09 ± 0.05	0.08 ± 0.04	0.408	0.667
C8	0.07 (0.05,0.11)	0.07 (0.04,0.12)	0.05 (0.04,0.09)	1.907	0.385
C10	0.07 (0.04,0.11)	0.05 (0.04,0.09)	0.05 (0.04,0.08)	1.128	0.569
C14	0.05 ± 0.02	0.05 ± 0.03	0.05 ± 0.03	0.025	0.975
C14:1	0.07 ± 0.04	0.07 ± 0.05	0.07 ± 0.02	1.709	0.425
C16	0.86 ± 0.24	0.84 ± 0.3	0.81 ± 0.3	0.2	0.819
C16:1-OH	0.05 ± 0.01	0.06 ± 0.04	0.06 ± 0.03	0.948	0.394
C16-OH	0.03 ± 0.02	0.03 ± 0.03	0.04 ± 0.02	1.933	0.154
C18	0.48 ± 0.13	0.62 ± 0.28	0.57 ± 0.22	2.761	0.252
C20	0.02 (0.02,0.02)	0.02 (0.01,0.03)	0.01 (0.01,0.03)	2.221	0.329
C24	0.04 ± 0.02	0.05 ± 0.02	0.04 ± 0.02	0.831	0.441
C26	0.03 (0.02,0.05)	0.03 (0.03,0.05)	0.03 (0.02,0.03)	0.682	0.711

* It compared with H1 group, *P*<0.05; It compared with H2 group, *P*<0.05.

**Abbreviation**s: C0, free carnitine; C2, acetylcarnitine; C3, propionylcarnitine; C4, butyrylcarnitine; C4-OH, 3-hydroxylbutyrylcarnitine; C4DC, succinyl-/methylmalonylcarnitine; C5, cisovalerylcarnitine; C5-OH, ovalerylcarnitine; C5DC, glutarylcarnitine; C5:1, tiglylcarnitine; C6, hexanoylcarnitine; C8, octanoylcarnitine; C10, decanoylcarnitine; C12, lauroylcarnitine; C14, myristoylcarnitine; C14-OH, 3-hydroxyl-tetradecanoylcarnitine; C14DC, tetradecanoyldiacylcarnitine; C14:1, tetradecenoylcarnitine; C16, palmitoylcarnitine; C16-OH, hydroxypalmitoylcarnitine; C16:1-OH, hydroxypalmitoleylcarnitine; C18, octadecanoylcarnitine; C20, arachidic carnitine; C22, behenic carnitine; C24, tetracosanoic carnitine; C26, hexacosanoic carnitine.

### The analysis of clinical data and metabolites

**Figure 2:**
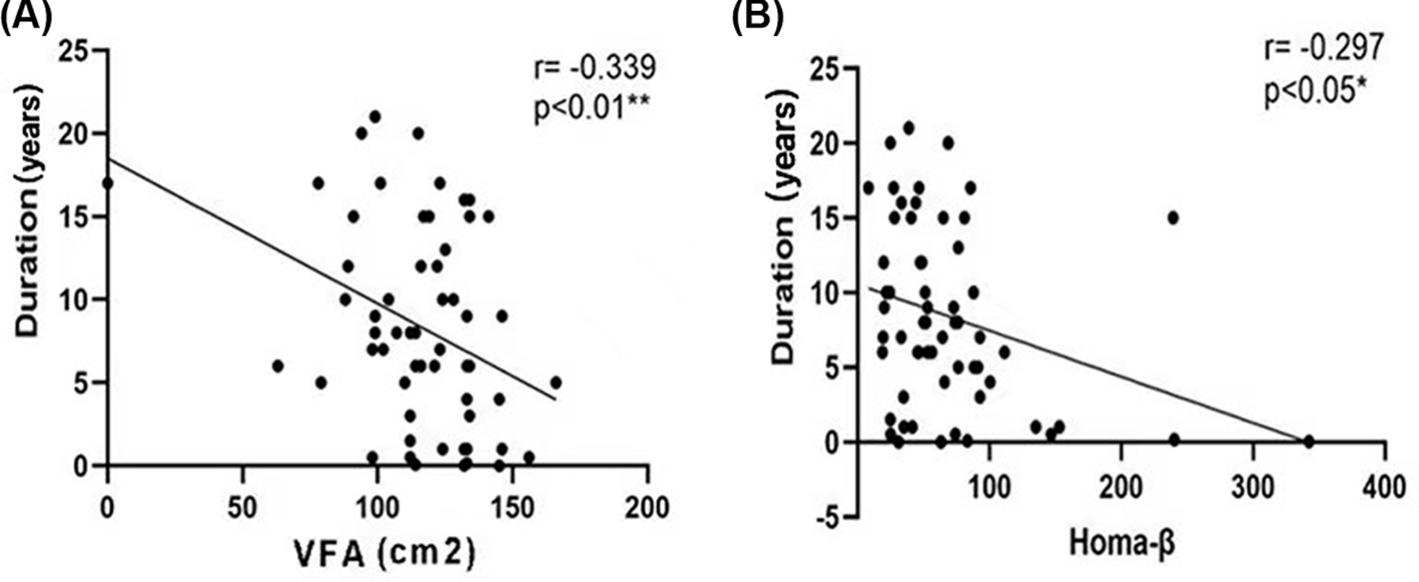
Correlation analysis of VFA, HOMA-β, and diabetes duration In overweight patients with T2D, the diabetes duration was negatively correlated with the VFA (**A**) and HOMA-β (**B**). ** The correlation was significant at a confidence level of 0.01; * the correlation was significant at a confidence level of 0.05; *R*: Spearman’s rank correlation coefficient

**Figure 3:**
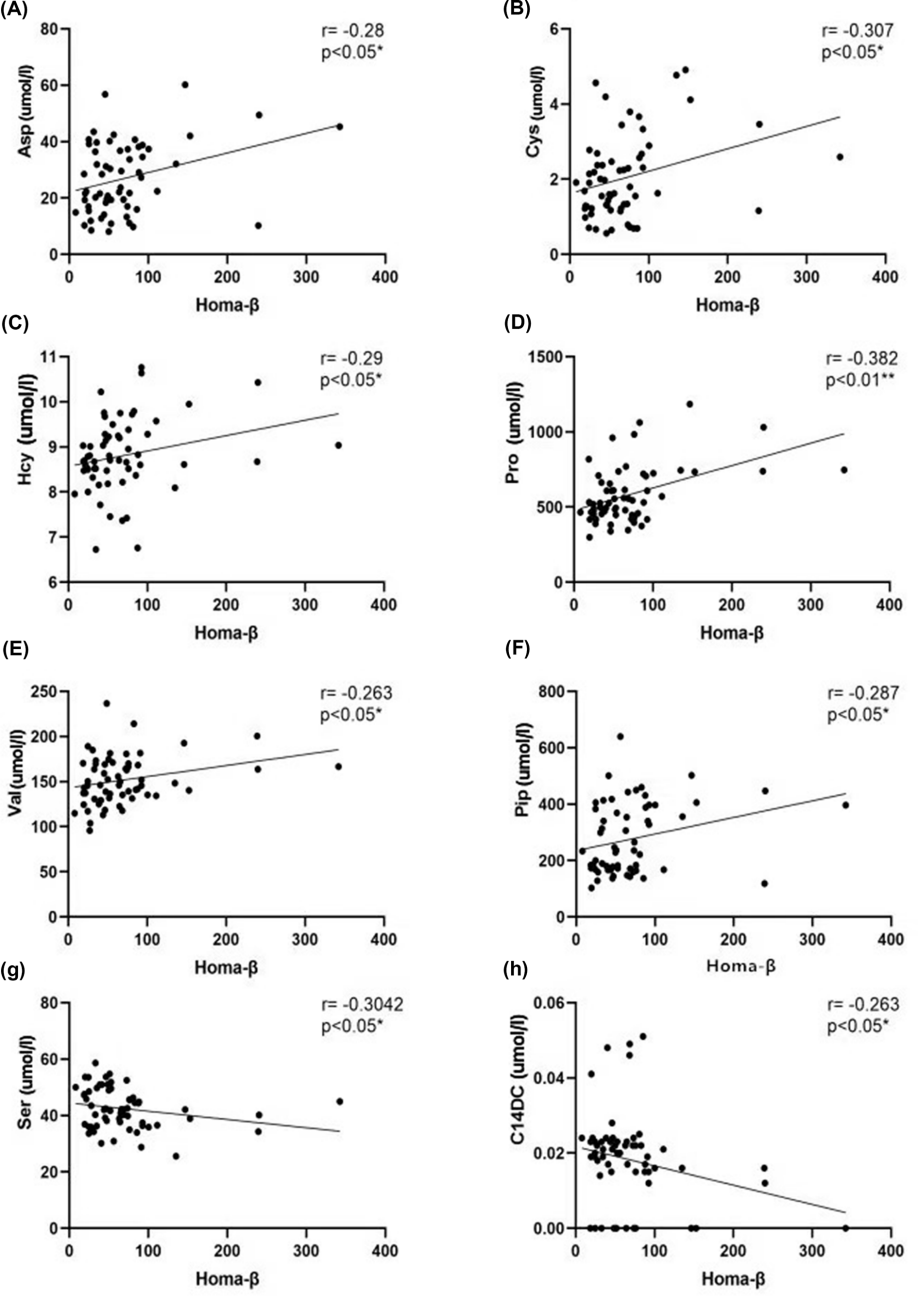
Correlation analysis of HOMA-β and metabolites In overweight patients with T2D, HOMA-β was positively correlated with the levels of amino acids including Asp, Cys, Hcy, Pro, Val, and Pip (**A–F**) and negatively correlated with Ser (**G**), and it was negatively with the level of acylcarnitine C14DC (**H**). ** The correlation was significant at a confidence level of 0.01; * the correlation was significant at a confidence level of 0.05; *R*: Spearman’s rank correlation coefficient.

**Figure 4:**
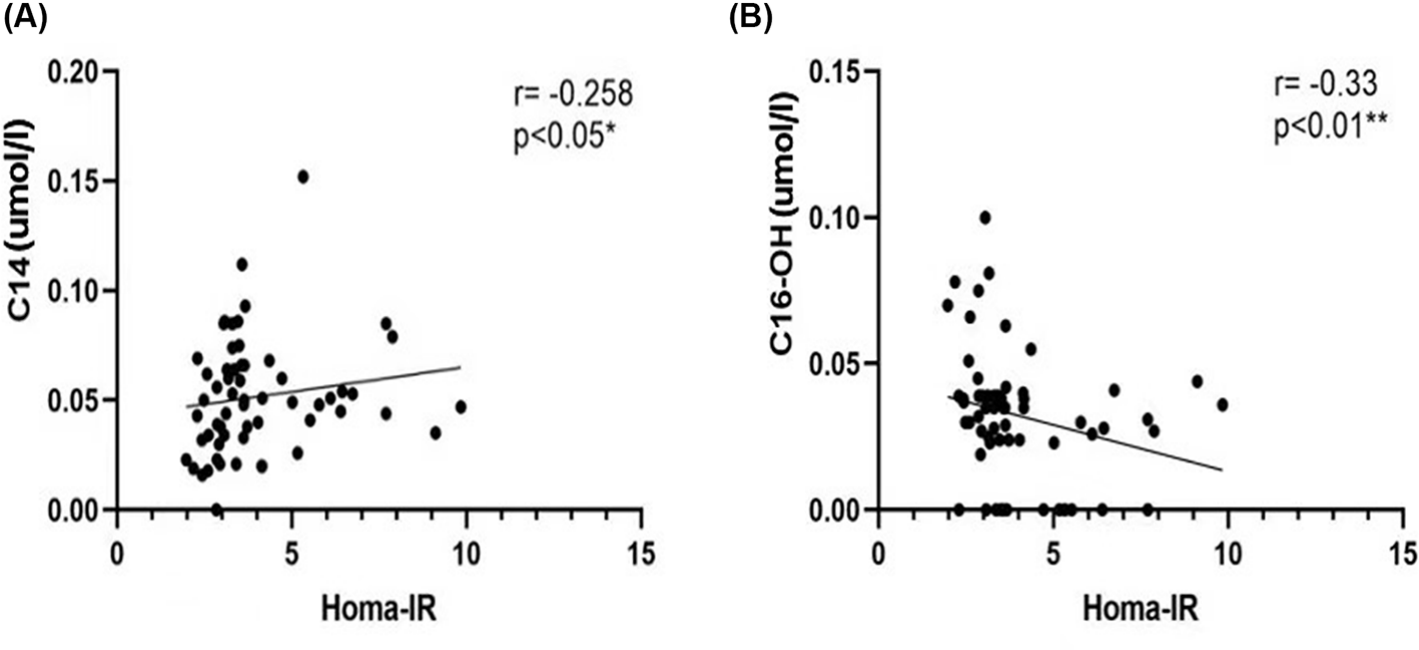
Correlation analysis of HOMA-IR and metabolites In overweight patients with T2D, HOMA-IR was positively correlated with C14 (**A**) and negatively correlated with C16-OH (**B**). ** The correlation was significant at a confidence level of 0.01; * the correlation was significant at a confidence level of 0.05; *R*: Spearman’s rank correlation coefficient.

To clarify the role of metabolomics in overweight patients with T2D, we analyzed the correlation between the clinical data and metabolites. The duration of diabetes was negatively correlated with the VFA and HOMA-β (Figure 2). The correlation analysis of the HOMA index and the levels of amino acids and acylcarnitine showed that HOMA-β was positively correlated with the Asp, Cys, Hcy, Pip, Pro, and Val levels, and was negatively correlated with the Ser and C14DC levels (Figure 3). HOMA-IR was positively correlated with the C14 levels and was negatively correlated with the C16-OH levels (Figure 4). The HOMA index was not correlated with other metabolites (Table 4).

**Table 4:** Correlation analysis of HOMA-index and metabolites

Metabolites	HOMA-β		HOMA-IR	
	*R*	*P*	*R*	*P*
Ala	−0.042	0.752	--0.073	0.577
Arg	0.2	0.125	0.174	0.183
Asn	−0.005	0.969	0.113	0.391
Asp	0.280*	0.03	0.136	0.3
Cit	--0.119	0.363	−0.145	0.268
Cys	0.307*	0.017	0.147	0.264
Gln	0.203	0.12	0.06	0.651
Glu	0.039	0.766	0.056	0.669
Gly	0.073	0.579	--0.031	0.816
Hcy	0.290*	0.024	0.022	0.866
His	0.23	0.077	0.153	0.243
Leu	0.237	0.068	0.238	0.067
Lys	0.179	0.17	0.043	0.746
Met	−0.02	0.88	0.041	0.758
Orn	0.111	0.396	0.052	0.694
Phe	0.081	0.54	0.168	0.199
Pip	0.287*	0.026	0.167	0.201
Pro	0.382	0.003	0.1	0.448
Ser	--0.304*	0.018	−0.011	0.934
Thr	0.094	0.476	0.044	0.74
Trp	−0.165	0.209	--0.03	0.821
Tyr	--0.075	0.567	−0.112	0.394
Val	0.263*	0.043	0.147	0.261
C0	0.053	0.685	0.107	0.415
C2	--0.008	0.953	0.017	0.897
C3	0.138	0.294	−0.011	0.932
C4	0.198	0.13	--0.049	0.713
C4-OH	0.119	0.363	0.009	0.946
C4DC	0.096	0.464	−0.039	0.766
C5	0.124	0.343	--0.059	0.656
C5-OH	0.039	0.767	−0.104	0.428
C5DC	−0.199	0.126	--0.048	0.716
C5:1	--0.221	0.089	−0.162	0.216
C6	0.152	0.247	--0.008	0.954
C8	--0.092	0.483	−0.093	0.479
C10	−0.067	0.609	--0.176	0.178
C12	--0.214	0.1	−0.216	0.097
C14	0.135	0.304	0.258*	0.047
C14-OH	−0.024	0.856	0.153	0.244
C14DC	--0.263*	0.042	−0.226	0.082
C14:1	−0.129	0.326	--0.046	0.727
C16	--0.066	0.617	−0.181	0.167
C16-OH	−0.186	0.154	--0.330	0.01
C16:1-OH	--0.012	0.927	−0.026	0.845
C18	−0.126	0.338	--0.233	0.074
C20	--0.108	0.412	0.007	0.958
C22	−0.11	0.404	--0.125	0.341
C24	--0.136	0.302	−0.017	0.896
C26	−0.091	0.491	--0.097	0.459

The correlation was significant at a confidence level of 0.01; * The correlation was significant at a confidence level of 0.05; *R*: Spearman’s rank correlation coefficient.

**Figure 5:**
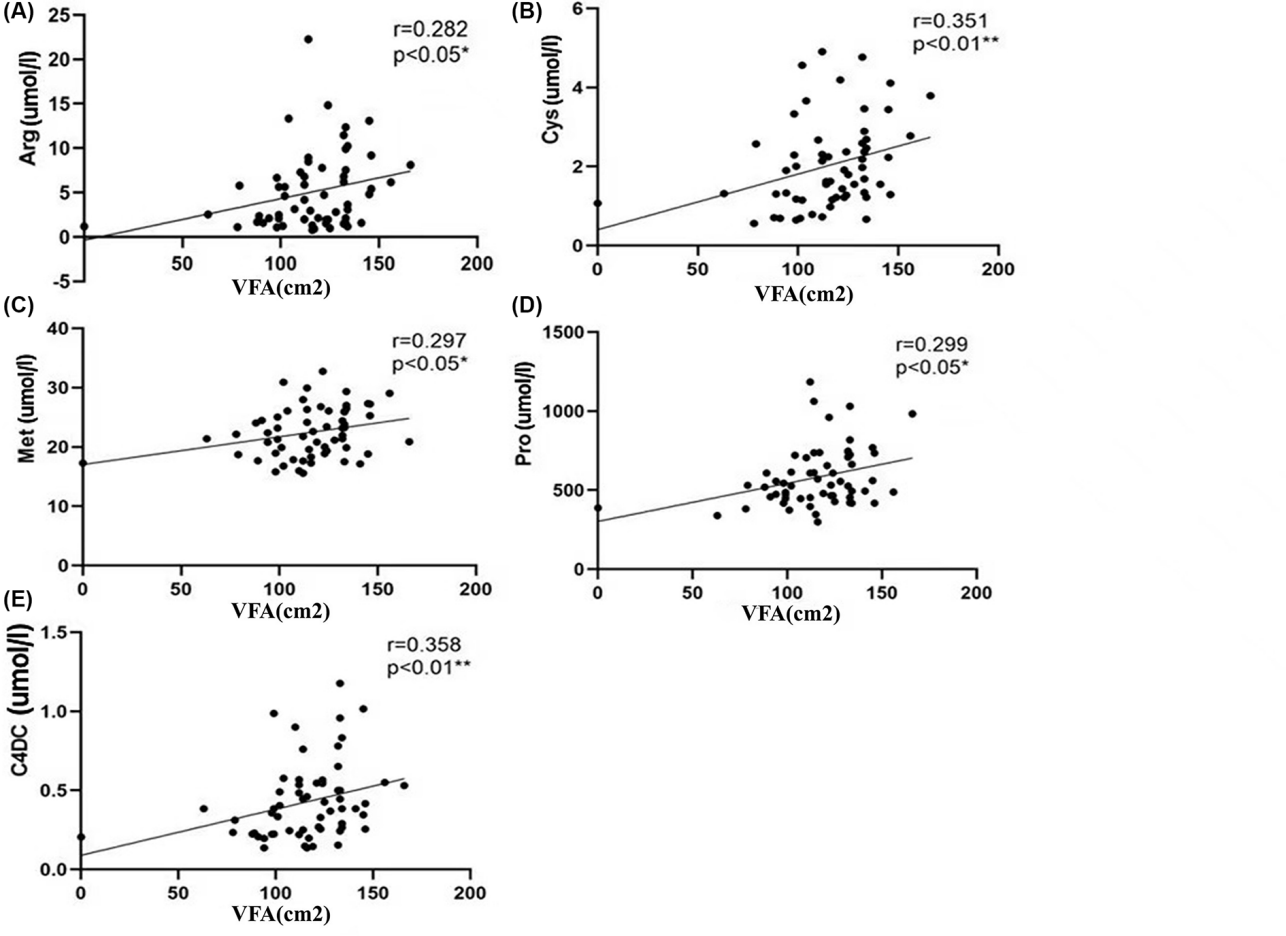
Correlation analysis of VFA and metabolites In overweight patients with T2D, VFA was positively correlated with the levels of amino acids including Arg, Cys, Met, and Pro (**A–D**) and the level of acylcarnitine C4-DC (**E**). ** The correlation was significant at a confidence level of 0.01; * the correlation was significant at a confidence level of 0.05; *R*: Spearman’s rank correlation coefficient

Being overweight and obese are common phenotypes of metabolic diseases. The VFA is an important index for the evaluation of obesity. In overweight patients with Type 2 DM, the correlation analysis between the VFA and the levels of metabolites showed that the VFA was positively correlated with the Arg, Cys, Met, Pro, and C4-DC levels (Figure 5).

## Discussion

The main pathophysiological disturbances in T2D are impaired insulin secretion and insulin resistance. It has been demonstrated that islet β-cell function deteriorates with the duration of diabetes. It is especially important to identify early biomarkers of β-cell function in T2D. Recent studies have highlighted the significance of metabolomics when studying metabolic diseases. We aimed to identify metabolites associated with islet β-cell function in overweight patients with T2D.

As early as 2011, Wang et al. [15] used liquid chromatography and tandem mass spectrometry to identify metabolites in plasma to predict the risk of T2D in healthy individuals. They found that five BCAAs and aromatic amino acids (isoleucine, leucine, valine, tyrosine, and phenylalanine) were significantly associated with T2D risk [15]. BCAAs, nonesterified fatty acids, and lysophosphatidylinositol are significantly associated with diabetes risk in Chinese adults [16]. Fasting metabolite profiles have been shown to distinguish T2D patients from individuals with normal glucose tolerance, and Li-Gao et al. revealed that both postprandial metabolites and fasting status could identify T2D equally [17]. Recently, total dairy consumption was found to be associated with metabolite levels and showed an inverse association with T2D risk [18]. Zhou et al. reported that differences in plasma amino acid concentrations were obvious in diabetic patients compared with nondiabetic subjects; these alterations were correlated with blood glucose, lipids, insulin, and hemoglobin status [19]. In older adults, diabetic patients had higher alanine, glutamate, proline, and lower arginine and citrulline levels than nondiabetic patients. Amino acids were also associated with cardiovascular functions in aging, independent of diabetes status [20]. Four amino acids, asparagine, aspartic acid, glutamine, and glutamate, and three carbohydrates, 1,5-anhydroglucose, fructose, and inositol, were significantly different between diabetic and nondiabetic patients [21]. These metabolites belong to the amino acid, energy, carbohydrate, and lipid metabolism pathways, which are known to be closely related to insulin resistance and secretion [22–25]. An increasing number of studies have confirmed a correlation between metabolomics and the occurrence and development of DM.

Glutamine/glutamate metabolism is associated with a variety of cellular functions such as protein synthesis, muscle growth, liver urea production, insulin secretion, gluconeogenesis, neurotransmitter synthesis, and glutathione production [26]. In particular, the regulation of insulin secretion by islet β cells has been considered a target for diabetes treatment [27]. In β cells, glutamine is transported by the blood and accumulates on the plasma membrane where it is converted to glutamate, which regulates insulin secretion through many coupling mechanisms between various molecules and enzymes [28]. Cheng et al. observed that plasma glutamine, glutamate, and the glutamine/glutamate ratio were closely related to insulin resistance in the Framingham Heart Study and the Malmö Diet and Cancer Study [29]. Rhee et al. [21] found that plasma glutamine and glutamate are potential biomarkers for predicting diabetic retinopathy, and that the significant differences in metabolites may be related to the duration of diabetes. In the study, the diabetes history of patients with and without retinopathy was more than 20 years, and metabolic changes were sustained in long-term chronic disease [21].

Our study analyzed the correlation between metabolites and HOMA-β in overweight patients with T2D. It has been proposed that the metabolic pathways of glutamine/glutamic acid and asparagine/aspartic acid may be related to the regulation of insulin secretion and resistance. Our study showed that aspartic acid levels were correlated with HOMA-β, which is consistent with previous study findings. In addition, we found that some amino acids (Cys, Hcy, Pip, Pro, Val, and Ser) were correlated with HOMA-β. Therefore, these amino acid metabolites are expected to serve as early biomarkers for the evaluation of islet β-cell function. There was no difference between HOMA-IR and amino acid metabolites, which was inconsistent with previous studies. This could be due to the population in this study being overweight with T2D, and insulin resistance in overweight with T2D was not obvious compared with obese patients.

Acylcarnitine is an intermediate metabolite involved in oxidative stress. Fatty acids are activated to acyl-CoA, which is converted into acylcarnitine and subsequently transported into the mitochondria for complete β-oxidation [30]. Elevated circulating levels of free carnitine, short-chain acylcarnitines (C2, C3, C4, C5), medium-chain acylcarnitines (C6, C8, C10, C10:1), and long-chain acylcarnitines (C14:1, C16, C18, C18:1) have been reported in T2D. Additionally, long-chain acylcarnitine is associated with the development of peripheral insulin resistance [30–33]. It is not clear whether acylcarnitine levels directly affect islet β-cell function. Recent studies have shown that accumulation of long-chain acylcarnitine, especially stearyl carnitine, impairs insulin synthesis [34], suggesting a potential link between altered acylcarnitine levels and β-cell dysfunction. When the production of acetyl-CoA exceeds the processing capacity of the tricarboxylic acid cycle, the level of medium-chain acylcarnitine increases quicker than that of long-chain acylcarnitine [24,32,35]. Therefore, altered levels of medium-chain acylcarnitine may be an early sign of mitochondrial dysfunction, possibly leading to β-cell dysfunction and the onset of DM. However, the exact mechanism is not clear. Batchuluun et al. [36] explored the effects of acylcarnitine on β-cell function in gestational DM and newly diagnosed T2D. Their results suggested that elevated medium-chain acylcarnitine levels are associated with β-cell dysfunction. The pathological concentrations of medium-chain acylcarnitine reduced the rate of glucose-stimulated ATP production in β cells, causing calcium influx damage and ultimately leading to decreased insulin secretion. The pathological concentrations of medium-chain acylcarnitine also cause functional changes in the mitochondria [36].

In the present study, we analyzed the correlation between HOMA index and metabolites in overweight patients with T2D. We found that the long-chain acylcarnitine C14DC was negatively correlated with HOMA-β, and that the level of C14DC increased with a decrease in β-cell function. Therefore, C14DC is a novel biomarker for evaluating pancreatic β-cell dysfunction. Correlation analysis between HOMA-IR and metabolites showed that HOMA-IR was positively correlated with C14 and negatively correlated with C16-OH, whereas previous studies suggested that acylcarnitine with different carbon lengths might have different effects on insulin resistance [37]. However, further studies are required to confirm this.

It is known that obesity is caused by complex interactions between genetic predispositions and environmental factors. However, the underlying mechanisms associated with abnormal metabolic phenotypes in obese individuals remain unclear. Several studies have examined the metabolite profiles associated with obesity according to metabolomics. Phospholipid metabolites have been identified as potential biomarkers for obesity-associated insulin sensitivity [38]. Elevated plasma levels of BCAAs and acylcarnitines have been associated with metabolic diseases. Lee et al. [39] observed different metabolites in obese individuals compared with normal-weight individuals. They found that higher levels of BCAAs and acylcarnitine, and lower levels of acyl-alkyl phosphatidyl choline were observed in obese children [39]. BCAAs may serve as early biomarkers for predicting metabolic diseases. Jourdan et al. [40] found that the composition of serum metabolites was closely related to obesity. The concentrations of BCAAs, the ratio of BCAAs to glycogenic amino acids, and an increased carnitine level were significantly correlated with an increase in fat-free weight. Allam-Ndoul et al. compared the differences in metabolomic characteristics between normal weight and overweight/obese individuals with or without metabolic syndrome. They found that amino acids and short- and long-chain acylcarnitines, including (C0, C16, C18, C3, C4, glutamate, isoleucine, leucine, methionine, phenylalanine, tyrosine, and valine) were positively correlated with HDL-C, and negatively correlated with insulin levels in normal weight individuals [41]. The above evidence suggests that metabolomics can be used as a predictor of obesity.

VFA is an important index for the evaluation of obesity. In the present study, we found that VFA decreased with disease progression in overweight patients with T2D. Arg, Cys, Met, Pro, and C4-DC levels were positively correlated with VFA. It has been found that metabolites such as short-chain acylcarnitine and BCAAs were correlated with obesity. In our study, C4-DC and Met were positively correlated with VFA, consistent with the results of previous studies. However, there are no previous reports on the correlation between Arg, Cys, Pro, and obesity, showing the novelty of the study.

## Conclusion

In summary, these findings suggest that serine, aspartic acid, cysteine, homocysteine, piperamide, proline, valine, and tetradecanoyldiacylcarnitine levels are associated with HOMA-β at different stages of T2D progression in overweight patients. They could, therefore, act as potential novel biomarkers for evaluating islet β-cell function.

## Data Availability

All supporting data are available from the corresponding authors.

## Abbreviations

BCAA: branched-chain amino acid
BMI: body mass index
HOMA: homeostasis model of assessment
IFG: impaired fasting glycemia
SM: sphingomyelin
T2D: Type 2 diabetes
VFA: visceral fat area
